# Supplementary material for: The Src-Family Kinases SRC and BLK Contribute to the CLDN6-Adhesion Signaling
Source: Cells. 2023 Jun 23;12(13):1696. doi: 10.3390/cells12131696 (PMC10341166; doi:10.3390/cells12131696)
Supplement: Supplementary file 1 [file cells-12-01696-s001.zip › cells-2426447-supplementary.pdf]

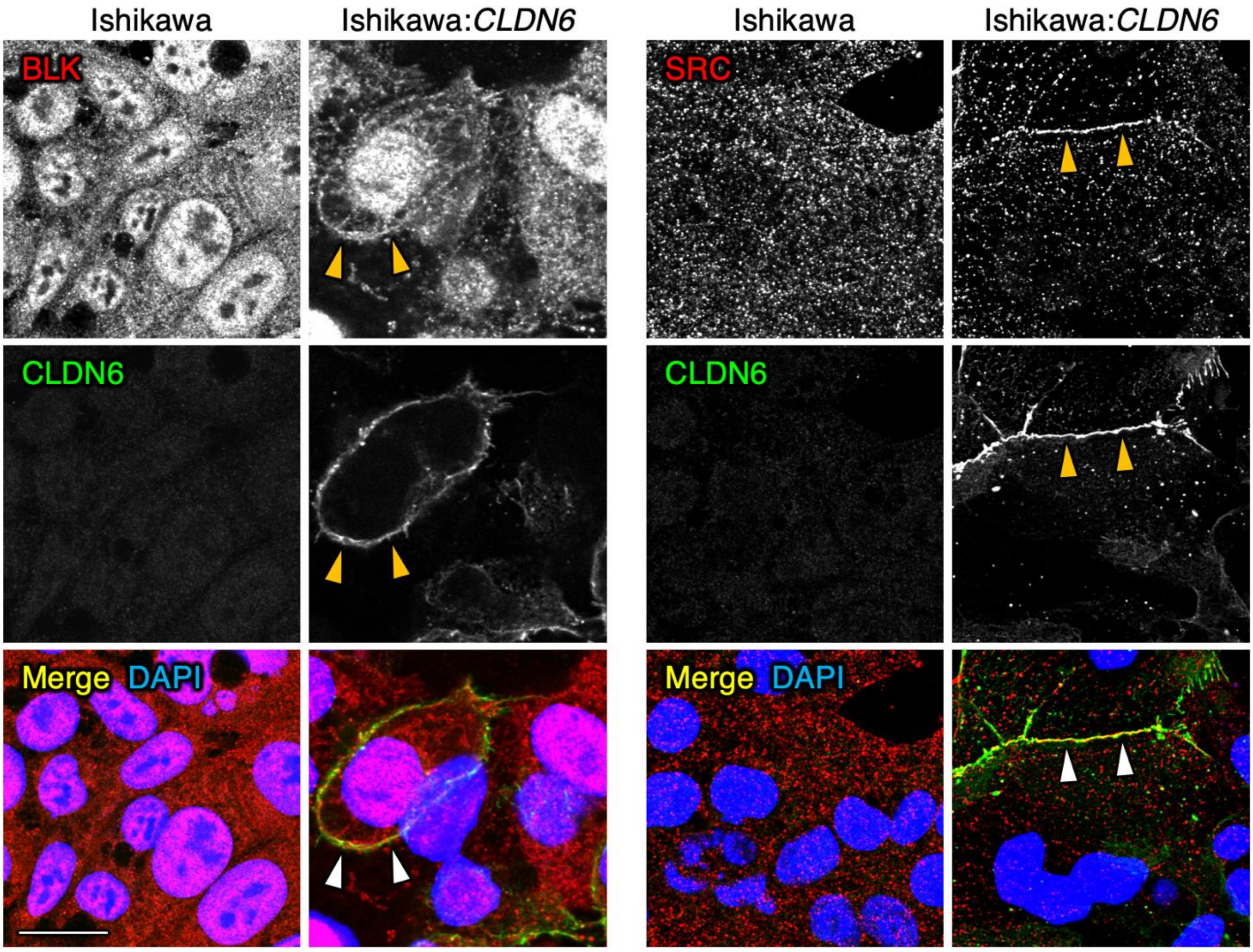

Supplementary figure S1. BLK and SRC are at least in part colocalized with CLDN6 along cell boundaries in Ishikawa:*CLDN6* cells but not in Ishikawa cells. Confocal images of indicated proteins in the revealed cell lines. Arrowheads indicate colocalization of SFKs and CLDN6. Bars, 20 µm.
